# The Cardiohepatic Axis in Heart Failure

**DOI:** 10.1016/j.jacbts.2025.05.007

**Published:** 2025-07-28

**Authors:** Arick C. Park, Joel D. Schilling

**Affiliations:** a Department of Internal Medicine, Washington University School of Medicine in St Louis, St. Louis, Missouri, USA; b Department of Pathology and Immunology, Washington University School of Medicine in St Louis, St. Louis, Missouri, USA

**Keywords:** biliary metaplasia, heart failure, liver fibrosis, macrophage, stellate cells

## Abstract

Cardiogenic liver disease is a sequela of hepatic congestion from right heart failure that leads to hepatic dysfunction and adverse liver remodeling. Understanding the mechanisms and consequences of liver pathology in the syndrome of heart failure is an important unmet need in the care of patients with chronic heart failure. Liver congestion provokes profound responses in hepatocytes and nonparenchymal cells including endothelial cells, macrophages, and hepatic stellate cells. However, the pathways that drive liver fibrosis and dysfunction are poorly understood. Recent human and preclinical data has begun to unravel the cellular landscape and molecular pathways that define cardiogenic liver disease. This review aims to discuss our current understanding of the cardiohepatic axis in heart failure while also highlighting knowledge gaps and opportunities for future investigation.

## Highlights

• Crosstalk between the heart and liver chronic heart failure is a complex process that involves hemodynamic and paracrine mechanisms.
• The cellular and molecular pathways that contribute to cardiogenic liver disease are only partially understood.
• Dissecting the cardiohepatic axis in heart failure requires a multifaceted approach that employs both patient samples and preclinical models.
• There is opportunity to discover novel biomarkers and therapeutic strategies to diagnose and manage cardiogenic liver disease.

Heart failure (HF) affects >6 million individuals in the United States and is estimated to exceed 8 million individuals by the year 2030.^1^^,^^2^ It is now recognized that the crosstalk between organs contributes to the syndrome of HF. Despite the high prevalence of liver dysfunction in HF,^3^ our understanding of organ crosstalk in its infancy. As a result, this is largely an untapped field with the potential yield novel diagnostic and therapeutic approaches.

The phenomenon of interorgan crosstalk between the heart and liver was first postulated in 1840 by the French physician, Louis-Alfred Becquerel, who noted chronic passive congestion from HF produced cirrhosis, contemporarily referred to as *foie cardiaque*, a term that encompasses the more colloquial verbiage of hepatic congestion and cardiac cirrhosis.^4^^,^^5^ Right heart failure (RHF) caused by biventricular cardiomyopathy, tricuspid insufficiency, pulmonary hypertension, or constrictive pericarditis, promotes liver congestion and fibrosis leading to what we have previously termed cardiogenic liver disease (CLD).^6^^,^^7^ Patients with RHF are thought to develop CLD as a consequence of chronically elevated central and hepatic venous pressures, which are transmitted to the hepatic circulation and central veins leading to adverse liver remodeling. Histologically, this is evidenced by sinusoidal dilation and red blood cell extravasation with centrilobular to bridging fibrosis which can progress to cirrhosis. The presence of liver fibrosis in patients with RHF is associated with increased morbidity and mortality.^8^^,^^9^ Additionally, patients with end-stage HF and concomitant RHF have an increased risk of primary graft dysfunction after heart transplantation and gastrointestinal bleeding after left ventricular assist device (LVAD) implantation, suggesting persistent multisystemic dysfunction that may not resolve after correction of the cardiac disease.^10^^,^^11^ Similar to patients with RHF, individuals with single ventricle congenital heart disease who undergo the Fontan procedure also develop congestive liver disease which is referred to as Fontan-associated liver disease (FALD). The study of FALD has the potential to accelerate our understanding of the events that drive liver pathology in CLD. Likewise, patients with Fontan stand to benefit from interventions that improve liver health in the setting of congestion. Despite the clinical relevance of CLD, how elevated venous pressures mediate liver pathology and how hepatic dysfunction influences prognosis is poorly understood.

One important consideration is the impact of liver disease on the failing heart itself. The notion that primary liver disease might influence cardiac structure and function was first incepted in a landmark study of cirrhotic cardiomyopathy in 1953, which described the hyperdynamic and vasodilatory result of alcoholic cirrhosis on cardiovascular function.^12^ Later studies demonstrated that patients with cirrhosis have impaired contractility and heart rate augmentation when challenged with exercise, which was mechanistically attributed to a desensitization in myocardial β-adrenergic receptors.^13^^,^^14^ A more detailed description of how liver disease impacts the heart is included in accompanying paper, *The Cardiohepatic Axis in Cirrhosis*, which is a part of this compendium. In addition, metabolic dysfunction–associated steatotic liver disease (MASLD) continues to increase in prevalence and is currently the leading cause of end-stage liver disease (see also *The cardiohepatic axis in metabolic liver disease*, also included in this compendium).^15^ Patients with HF have a high prevalence of MASLD, but the impact of combined metabolic and congestive stress on liver disease is unknown.

The past 5 years have witnessed a renewed interest in the heart-liver axis in HF. This review will highlight our current understanding of CLD, including FALD, with an emphasis on the cellular and molecular players involved in liver remodeling. In addition, the systemic consequences and clinical implications will be discussed.

## Adverse Liver Remodeling in HF

### Hemodynamics

**Figure 1:**
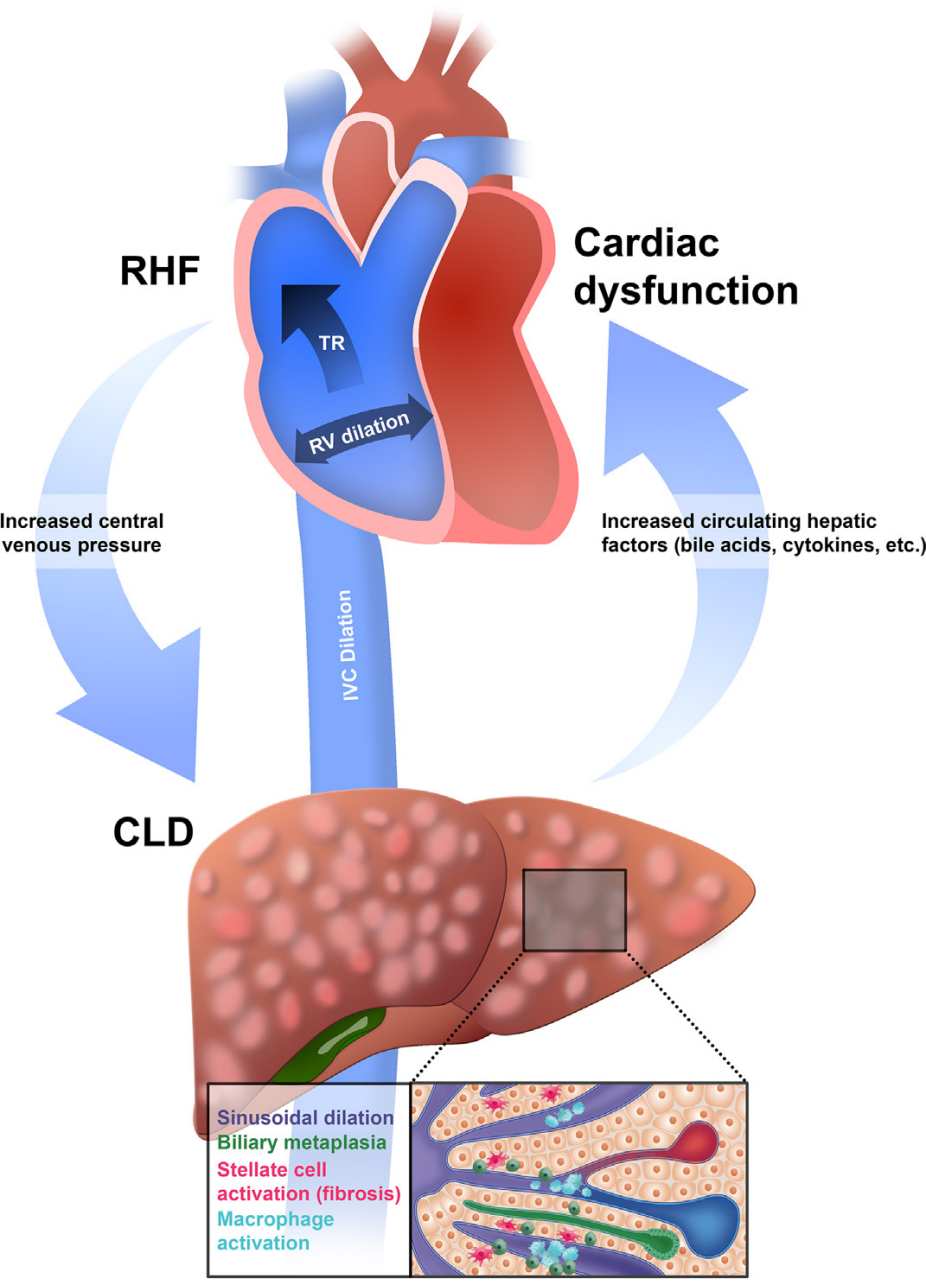
Bidirectional Crosstalk Between Heart and Liver in Heart Failure CLD = cardiogenic liver disease; IVC = inferior vena cava; RHF = right heart failure; RV = right ventricular; TR = tricuspid regurgitation.

Cardiogenic hepatic dysfunction is thought to result from the combination of decreased cardiac output and elevated central venous pressures (Figure 1). The syndrome of HF can cause both acute liver injury and chronic liver fibrosis and dysfunction. Acute ischemic hepatitis, or shock liver, results from hypoperfusion because of low cardiac output and presents with marked elevations in serum aminotransferase concentrations. Ischemic hepatitis is usually self-limited and improves when cardiac output is restored.^16^ The susceptibility of the liver to ischemic injury is heightened by liver congestion. This is likely caused by the liver’s dual blood supply, receiving both deoxygenated blood from the portal vein and oxygen-rich blood from the hepatic artery, which buffers the liver from acute hypoxic events.^17^ In shock liver, it is the reduced hepatic artery flow and elevated central venous pressures (CVPs) that effectively reduces the transhepatic pressure gradient further contributing to reduced clearance, hypoperfusion, and ischemia. This concept has been borne out in observational studies of patients with decompensated low-output HF where it was the combination of both elevated CVPs and reduced hepatic blood flow that characterized ischemic hepatitis.^18^^,^^19^

In contrast, chronic liver disease resulting from RHF is primarily driven by chronic elevations in CVP and hepatic venous pressures rather than low output. This congestive stress results in adverse liver remodeling, traditionally characterized by hepatic fibrosis. Supporting a primary role for venous congestion in CLD, rodent models of partial inferior vena cava ligation (pIVCL), which increases hepatic venous pressures, recapitulates many pathologic features of congestive hepatopathy in humans.^20^ These findings suggest that increased intrahepatic venous pressures more so than low output or paracrine factors drive CLD. However, the molecular pathways that connect increased hydrostatic pressures into adverse liver remodeling remains unclear. In the following sections, we will highlight several recent studies that have identified putative mechanisms of liver dysfunction in HF.

### Pathologic and cellular features

#### Sinusoidal dilation and vascular alterations

As mentioned in the previous text, the common theme linking RHF to liver remodeling is the chronic elevation of CVP. The increased hydrostatic pressures result in dilation of and hemorrhage into the sinusoids. Liver sinusoid endothelial cells, along with macrophages and hepatic stellate cells (HSCs), create a complex vascular network that regulates blood flow and filtration efficiency.^21^, ^22^, ^23^, ^24^ Sinusoidal dilation is associated with elevated right atrial pressures and serum bilirubin, further implicating congestion as an essential component in CLD (Figure 1).^22^ Additionally, our group and others have observed increased CD34-positive endothelial cells, a marker of sinusoidal activation, in liver biopsy samples from RHF patients.^7^ Importantly, increased CD34 expression has been associated with risk of hepatocellular carcinoma (HCC) in MASLD.^25^^,^^26^ Endothelial cells subjected to mechanical stress also release cytokines and chemokines. In a recent example, endothelial stretch in a mouse model of liver congestion led to the release of the chemokine CXCL1, which promoted neutrophil recruitment and NETosis.^27^ Additional investigative work is required to understand how increased hepatic venous pressures activate sinusoidal endothelial cells and contribute toward adverse hepatic remodeling.

#### Fibrosis and HSC activation

Fibrosis is a ubiquitous feature of CLD. Prior observational studies of liver pathology from patients with RHF have demonstrated that fibrosis tends to emanate from zone 3. However, periportal and bridging fibrosis is also observed.^21^, ^22^, ^23^, ^24^ In a recent study of patients with HF who underwent liver biopsy during the evaluation for advanced HF therapies, the prevalence of advanced fibrosis was 17%.^9^ Zone 3 encompasses the central hepatic veins, which are in closest proximity to elevated central venous pressures and therefore is first to manifest with pathologic changes. However, while the severity RHF plays a role in the degree of adverse liver remodeling, our group found that snapshot assessments of right atrial pressures or tricuspid regurgitation severity did not correlate with liver disease severity. Rather, the duration of HF was the strongest predicator of pathologic liver remodeling, implicating chronicity as a key factor in disease pathogenesis.^7^ This time-dependent phenomenon is also found in congenital heart disease patients who are palliated with a Fontan, where liver fibrosis is universal by 10 years following the operation.

HSCs are the primary collagen producing cell in the liver; however, portal fibroblasts also contribute to matrix deposition.^28^ HSCs are pericytes that reside in a quiescent state in the peri-sinusoidal space. Upon activation via injury or inflammatory signals, HSCs can take on inflammatory/senescent or myofibroblast phenotype (Figure 1). Our group found that HSCs expand and activate in liver samples from patients with RHF and mice that have undergone pIVCL. Additionally, transcript data from these mice confirmed elevated levels of *Acta2* and major fibrillar collagens.^7^ However, the upstream signals that activate HSCs in response to liver congestion are not well defined, although Activins A and B may contribute.^29^ The interplay among HSCs, endothelial cells, and macrophages requires more investigation.

#### Biliary metaplasia

Cholangiocytes are specialized epithelial cells of the bile canaliculi whose primary function is to transport bile acids from hepatocytes to the larger bile ducts. In response to damage, as occurs in primary biliary cirrhosis or primary sclerosing cholangitis, cholangiocytes proliferate and contribute to inflammation and fibrosis. This response has been referred to as the ductular reaction and is thought to be an adaptation to handle defective bile transport. Invasive ductular reaction occurs when cells expressing cholangiocyte markers (such as cytokeratins [CK]) appear outside the portal tracts in the liver parenchyma following injury. In this case, the CK-expressing cells are thought to arise from hepatocyte precursors via a process referred to as biliary metaplasia. The origin of the biliary metaplastic cells can include progenitor cells, cholangiocytes, or hepatocytes.^30^ In addition to the variable ontogeny of these cells, biliary metaplasia has been linked to both reparative, and/or pathologic responses in the liver.

Pathologic analysis of livers from patients with RHF has revealed that biliary metaplasia is a universal feature of CLD. As in other liver conditions, the extent of biliary metaplasia also correlated with other features of pathologic liver remodeling.^30^, ^31^, ^32^ Hepatocyte-progenitor cells (HPCs) are multipotent cells that express both hepatocyte- and biliary epithelial-specific markers, including hepatocyte nuclear factor 4α and CK, respectively. Following liver or bile duct injury, HPCs can differentiate into either hepatocytes or biliary endothelial cells to facilitate regeneration.^33^, ^34^, ^35^ In the context of CLD, our group and others have observed expansion of these CK-expressing metaplastic cells in the liver parenchyma. We further demonstrated that these metaplastic cells coexpress hepatocyte nuclear factor 4α and CK7 suggesting they arise from HPCs or dedifferentiated hepatocytes.^7^^,^^36^^,^^37^ Compared with other chronic liver diseases, the extent of invasive ductular reaction in more pronounced in congestive liver samples. Although the functional consequence of biliary metaplasia in CLD is unknown, the fact that this reaction colocalizes with activated HSCs and collagen rich regions suggests a role in adverse liver remodeling. Additional investigation is necessary to understand the upstream signals and downstream sequalae of biliary metaplasia in hepatic congestion.

#### Macrophage biology

Innate immune cell activation is a common feature in most chronic liver diseases including MASLD, alcoholic hepatitis, viral hepatitis, and autoimmune hepatitis.^38^^,^^39^ In contrast, pathologic studies from liver tissue obtained from patients with RHF has suggested that CLD is not an inflammatory process. However, this statement is largely based on the lack of obvious immune cell aggregates on hematoxylin and eosin stained sections.^4^^,^^20^^,^^40^ The liver is the largest reservoir of resident macrophages in the human body. These resident macrophages, known as Kupffer cells (KCs), are essential to maintaining homeostasis and are frequently perturbed in disease states. For example, metabolic liver disease and carbon tetrachloride toxicity are associated with KC loss and infiltration of monocyte-derived macrophages (MdMs).^41^, ^42^, ^43^ Our group recently characterized the impact of hepatic congestion on liver macrophages. In contrast to other liver diseases, resident KC numbers actually increased in number in response to congestion. Moreover, KC cell size and the number of macrophage aggregates was increased in both livers from patients with HF or mice that had undergone pIVCL.^7^^,^^44^ Macrophages in the aggregates lacked expression of the canonical KC marker MARCO, suggesting they may represent “reprogrammed” KCs vs MdMs that entered the liver upon injury. The lipocalin LCN2 is associated with inflammation and fibrosis and is rarely expressed in the normal liver. However, in congested livers the number of macrophages that are LCN2 positive increases dramatically (Figure 1).^7^ LCN2 expression is induced by exposure of macrophages to low-dose LPS or dead hepatocytes, both of which are prevalent in CLD.

Although monocyte recruitment occurs in response to liver congestion, it is relatively modest. Thus, KC responses are likely the main driver of liver inflammation during the progression of CLD. At present, the triggers of KC activation with congestion are speculative but could include excessive iron from increased erythrocyte uptake, mechanical stretch, microbial antigens from the intestine, or antigens released from injured or dying hepatocytes. In future investigation, it will be important to determine the adaptive and maladaptive features of KC activation and whether these pathways can be harnessed to reduce liver damage and fibrosis.

### Molecular pathways involved in liver remodeling

#### Thrombosis

The mechanisms by which hemodynamic and mechanical stress translate to adverse liver remodeling remain unclear. One interesting study demonstrated that sinusoidal dilation was associated with fibrin deposition and thrombosis in liver samples from patients with HF and in rodents that have undergone pIVCL surgery. Because HSCs can be activated by stretch and contribute to assembly of fibronectin fibrils, the investigators sought to determine if targeting platelet thrombus formation would impact liver remodeling. Treatment with either warfarin or through transgenic overexpression of tissue factor pathway inhibitor attenuated congestion-induced liver pathology.^20^ Other groups have also implicated fibronectin in pIVCL hepatic disease progression.^45^, ^46^, ^47^ Many patients with HF are already on anticoagulation, but little is known about the impact on liver outcomes. The role for anticoagulation in the treatment of CLD remains unclear, especially given the potential increased risk of bleeding in these patients.

#### Hypoxia

Chronic hypoxia, as a consequence of either low cardiac output or persistent congestion from RHF may contribute to liver fibrogenesis and fibrosis. Both hypoxic stress and pIVCL-induced hepatic congestion promotes hypoxia-inducible factor (HIF) transcription factors mRNA and protein expression.^47^^,^^48^ Hypoxia has also been shown to activate HSCs toward a myofibroblast phenotype. In other liver disease models, such as bile duct ligation (BDL), the targeted deletion of *hypoxia-inducible factor 1-alpha* in hepatocytes and HSCs reduced fibrosis in mice.^49^ Similar results have also been found in mice with a myeloid-specific knockout of HIF transcription factors, where BDL-induced fibrosis was again reduced.^50^ Whether HIF signaling contributes to hepatic fibrosis CLD has not yet been investigated.

#### Inflammation

The liver is an enriched with immunologic cells including a high density of macrophages (see the previous section), which can contribute to local and systemic inflammation. Our group recently explored the inflammatory milieu in patients with RHF compared with patients with either left HF or control subjects. Patients with RHF had higher serum concentrations of KC-derived cytokines, including soluble CD163 (sCD163) and CXCL12.^44^ Other investigators have also found that the level of sCD163 is associated with of KC activation, progression of hepatic fibrosis, and development of HCC in patients with liver disease.^51^^,^^52^ However, it is unclear if sCD163 contributes to disease pathogenesis and/or serves as a prognostic biomarker. In the human liver CXCL12 expression is also substantially enriched in KCs and its expression is up-regulated by hypoxia in a HIF-dependent manner where it may promote HSC activation.^53^^,^^54^ Additional studies are warranted to determine the precise role of CXCL12 in the pathogenesis of CLD.

Infiltration of MdMs into the liver is a common following hepatic injury and is often central to hepatic inflammation and fibrosis (Figure 1). In liver samples from RHF patients, our group observed macrophage aggregates lacking canonical resident KC markers, suggesting they may be monocyte derived. This was corroborated in pIVCL mice, which showed increased Ccr2-macrophages/monocytes in the congested liver.^7^ However, as discussed in the previous text, MdM recruitment in CLD is modest and whether these cells play a significant role in liver pathology remains to be determined.

#### Fontan-associated liver disease

Patients with single ventricle congenital heart disease often require transplant or Fontan procedure to survive. The Fontan circulation directly routes deoxygenated blood from the SVC and IVC to the pulmonary arteries. This circulation requires persistently elevated central venous pressure to drive blood flow. Thus, intrahepatic pressures are also elevated, which places congestive stress on the liver. Patients with Fontan circulation universally develop liver pathology which is referred to as Fontan-associated liver disease (FALD). FALD is associated with liver fibrosis, cirrhosis, HCC, and death.^55^, ^56^, ^57^ In many ways, FALD can be viewed as the penultimate example of CLD because of the persistent elevations in CVP that are sustained over time. As such, the investigation of FALD has the potential to improve the care of patients with living with a Fontan as well as provide insights into the mechanisms CLD more broadly.

The histopathology of FALD is similar to that described for patients with RHF, and includes sinusoidal dilation, centrilobular fibrosis, hepatocyte necrosis, and cirrhosis.^58^ Probably related to the chronicity of the liver congestion, patients with FALD are at increased risk for HCC.^59^ Recent studies have also begun to identify molecular pathways that may contribute to the pathogenesis of FALD. In a study by Bravo-Jaimes et al,^60^ they utilized a RNA sequencing (RNA-seq) approach on fixed liver tissue from over 100 patients with Fontan who had undergone liver biopsy and identified pathways related to cytokines, transforming growth factor-β, and vascular development were enriched in samples with advanced liver fibrosis.^53^ Of interest, another recently published study performed single nuclear RNA-seq analysis of liver biopsy tissue from patients with early stage FALD demonstrated profound changes in central vein hepatocyte gene expression. Based on these findings, the investigators suggest that central hepatocytes may serve as “first responders” to congestive stress by releasing activins, which promote HSC activation.^29^^,^^60^ Of note, the cellular response in FALD is distinct from metabolic dysfunction associated steatohepatitis, supporting the concept that CLD has a distinct pathology. Bridging the knowledge gap in adult CLD will help to inform putative mechanisms and therapies in FALD and vice versa.

#### Advanced HF therapies in patients with CLD

Heart transplant and/or LVADs can lead to substantial improvements in survival and quality of life in carefully selected patients with advanced HF. The presence of significant liver disease has been associated with worse perioperative outcomes, potentially related to increased bleeding risk, vasoplegia, and/or primary graft dysfunction.^10^^,^^11^ In cases of more advanced liver disease single organ transplantation may not be possible, although the criteria for determining the need for dual organ transplantation are not clear.^61^^,^^62^ This is particularly relevant for patients with FALD. Moreover, when considering LVAD the presence of liver pathology is associated with increased risk of right ventricular failure and mortality after implantation.^63^ In fact, significant liver remodeling may identify patients with chronic right ventricular failure that are unlikely to do well with LV only support. For these reasons, developing better biomarkers and imaging tools will be important to guide clinical decision making.

## Diagnosis of Cardiogenic Liver Disease

RHF is prevalent in patients with end-stage cardiomyopathies and is more frequent in those with nonischemic etiology and/or pulmonary HTN. Liver dysfunction and fibrosis is common in patients with RHF and is associated with worse outcomes. As mentioned in the previous text, the presence of advanced fibrosis and/or cirrhosis can be a contraindication for single-organ heart transplant or LVAD. Thus, accurately diagnosing liver pathology is essential.^64^, ^65^, ^66^ Although biopsy is the gold standard for the diagnosis of liver fibrosis, there is a lack of standard pathologic grading criteria for CLD as compared with other primary liver diseases.^67^ Clinicians often rely on clinical factors, imaging findings, and/or biochemical markers to inform the decision for biopsy. However, there is a lack of data to guide liver evaluation in patients with HF.

### Biochemical markers

Congestive hepatopathy caused by RHF often produces a cholestatic pattern of livery injury, predominantly reflected by an elevation of serum bilirubin and alkaline phosphatase. This may also be accompanied by a mild elevation in liver enzymes, AST, and ALT. Serum concentrations of bilirubin, AST, and ALT are predictive of mortality in patients with acute and chronic HF.^68^, ^69^, ^70^ For other chronic liver diseases including chronic hepatitis B, hepatitis C, and MASLD, the AST to platelet ratio index and fibrosis-4 scores have been developed to predict the risk of hepatic fibrosis. These scores include several hematological and biochemical values associated with liver pathology. However, when applied to patients with RHF, there is a poor correlation between these indices and the degree of hepatic disease.^7^ The Model for End-Stage Liver Disease Excluding INR (MELD-XI) score, which incorporates serum bilirubin, similarly had a weak correlation with liver pathology, r = 0.4, in patients with Fontan.^71^ Other potential surrogates of liver dysfunction and fibrosis include hepatically derived factors elevated in patients with RHF, such as bile acids, sCD163, and CXCL12. One of the challenges for the future will be to identify biomarkers that reflect chronic liver remodeling and not just acute congestion.

### Imaging

Liver ultrasound and computed tomography are common imaging modalities used to identify liver disease when there is clinical suspicion of hepatic pathology. Signs of liver disease can include heterogenous texture, surface nodularity, and/or stigmata of portal hypertension. However, several recent studies have shown a poor correlation between abnormal liver imaging findings and the extent of biopsy-proven fibrosis.^7^^,^^66^^,^^72^ More recently, liver stiffness measurements by either ultrasound or magnetic resonance elastography have been employed, but in the case of CLD, they are of limited utility because the results can be confounded by liver congestion.^73^ Developing specific fibrosis imaging modalities will be important to enhance the detection the noninvasive detection of liver pathology in patients with HF.

### Liver biopsy

Due to the lack of data correlating histologic fibrosis to invasive hemodynamics, biomarkers, or various imaging techniques, as described in the previous text, liver biopsy remains the gold standard and only reliable method for accurately diagnosing the extent of CLD and fibrosis. Despite this, liver biopsies have limitations including sampling error and a lack of standardized histological scoring. To the second point, a novel grading system was developed to grade liver fibrosis in patients with HF.^67^ However, whether this scoring system provides improved prognostic information for clinical decision making is unknown.

## Consequences of CLD

### Local effects

Pathologic liver remodeling and dysfunction is common in patients with end-stage HF. The prevalence of biopsy-proven fibrosis and cirrhosis was 94% and 15%, respectively, in patients with HF that were referred for liver biopsy.^7^ In patients with Fontan physiology, hepatic fibrosis is universal with a significant proportion progressing to cirrhosis.^55^^,^^58^ As a consequence, patients with FALD are more likely to develop portal hypertension and HCC.^74^ In patients with advanced RHF, clinically significant portal hypertension (hepatic venous pressure gradient >10 mm Hg) is a rare phenomenon. However, patients with FALD are more likely to present with sequelae of portal hypertension, including the presence of elevated VAST score (esophageal varices, ascites, splenomegaly, and thrombocytopenia). Patients with FALD and portal hypertension (elevated VAST score) have a 9-fold increased risk of major adverse events.^75^ HCC is a common complication of cirrhosis and requires regular surveillance. The pathogenesis of HCC is a complex multistep process that is driven by changes in tumor microenvironment, which includes immune cell reprograming and the altered metabolite milieu as seen in patients with CLD.^76^^,^^77^ In the context of FALD there is a 1.5% to 5% risk of developing HCC, which is significantly greater than the general population.^74^ Furthermore, the risk of developing HCC correlates with surrogate markers of portal hypertension and liver dysfunction.^78^

### Systemic effects

#### Impaired clearance

The liver plays a critical role in the metabolism and detoxification of exogenous and endogenous factors from portal and systemic circulation. Hepatic dysfunction and fibrosis results in impaired clearance of drugs, bacteria, and vasoactive peptides. The mechanism of impaired clearance is multifactorial. When hepatic circulation is decreased, either caused by low cardiac output or elevated portal pressures, there is theoretically less blood volume circulated per hepatocyte, the major cell type involved in drug metabolism. Furthermore, histologic evidence of hepatocyte necrosis and replacement fibrosis suggest there are fewer functional hepatocytes. In support of this concept, the expression of drug metabolizing enzymes is reduced in cirrhotic patients.^79^ Additionally, endothelial dysfunction and fibrosis are common in CLD. Capillarization of sinusoidal endothelial cells results in the loss of fenestrations, effectively preventing hepatocytes from metabolizing protein-bound drugs and larger vasoactive molecules.^80^ Similarly, dysfunctional sinusoids and KC dysfunction, as described above, can contribute to ineffective bacterial clearance. In animal models of liver fibrosis, KCs lose contact with parenchymal cells which also results in impaired filtration of the blood stream.^81^^,^^82^ The inability to clear potentially toxic substances from circulation can have systemic consequences leading to adverse events and complications.

#### Accumulation of metabolites and bile acids

Hepatic congestion leads to elevated levels of conjugated bilirubin and alkaline phosphatase in circulation, which mimics that seen in cholestatic liver disease. Increased bilirubin is also predictive of mortality in patients with HF.^68^, ^69^, ^70^ Bile acids are produced from cholesterol in hepatocytes and subsequently transported into the bile ducts. Chronic HF and FALD are associated with altered levels of serum bile acid concentrations, which are associated with poor survival and coronary artery disease.^83^, ^84^, ^85^ In addition to their role in digestion of lipids, bile acids are also signaling molecules via receptors such as farnesoid X receptor, Tekeda G protein coupled receptor 5, and sphingosine 1 phosphate receptor 2.^86^, ^87^, ^88^ Within the liver, elevated bile acids can activate hepatic MdMs and promote biliary epithelial proliferation, characteristic findings of CLD.^89^ Additionally, bile acids can have systemic consequences, including negative chronotropic effects on the heart (Figure 1).^90^, ^91^, ^92^

#### Lipoproteins

The liver synthesizes and packages lipoproteins which deliver cholesterol and triglycerides from the liver to other tissues. In cirrhotic patients, there is a marked decrease in low-density lipoprotein (LDL), high-density lipoprotein (HDL), and apolipoprotein (Apo)A-I.^93^^,^^94^ Similarly, patients with chronic HF also have lower LDL and total cholesterol compared with healthy control subjects and lower LDL is associated with worse survival.^95^^,^^96^ ApoM is an HDL-associated lipocalin primarily secreted by the liver and found in HDL and LDL. In addition to its anti-inflammatory and antiatherogenic effects, reduced levels of ApoM independently associate with mortality in patients with HF.^97^ Defects in lipoprotein quantity and composition reflect impaired liver function and may impact the nutrient supply for other tissues such as the heart and skeletal muscle.

### Impact on the syndrome of HF

#### Cardiac cachexia

Cardiac cachexia is common in patients with advanced HF and is associated with unintentional weight loss and skeletal muscle wasting. The pathogenesis is multifactorial, including impaired nutritional intake as well increased catabolism in several tissues.^98^ Patients with advanced HF commonly experience early postprandial satiety and intestinal malabsorption, which reduces overall caloric intake. Deficiencies in lipoproteins may also contribute to impaired shuttling of essential nutrients and signaling molecules between the liver and peripheral tissues. In the context of Fontan circulation, protein-losing enteropathy (PLE) also contributes to cachexia. PLE results from the combination of low cardiac output and elevated central venous pressures causing hepatic and enteric congestion. Cardiac cachexia is also associated with a proinflammatory milieu, which may impair myogenesis and increase catabolic activity. Growth differentiation factor 15 (GDF15) is a cytokine and independent predictor of HF disease, mortality, and cachexia.^99^^,^^100^ Similarly, patients with Fontan physiology have elevated levels of GDF15.^101^ In a mouse model of cardiac cachexia, GDF15 antagonism improved body weight, cardiac function, and cardiac fibrosis.^100^ These data implicate cytokines as both biomarkers of disease, but also as therapeutic targets for cachexia. Whether the liver is primary producer of GDF15 in HF remains to be determined.

#### Worsening heart function and increased arrhythmias

Cardiac dysfunction driven by cirrhosis, termed cirrhotic cardiomyopathy, is a well-described phenomenon that results in sympathetic overactivity, hyperdynamic cardiac output, splanchnic vasodilation, reduced systemic vascular resistance, and a blunted cardiac response to exercise.^13^^,^^14^ Multiple studies with both patients and rodent models of cirrhosis have attributed the lack of cardiac response to β-adrenergic receptor desensitization, reduced receptor density, and diminished downstream cAMP signaling.^13^^,^^14^^,^^102^^,^^103^ When challenged with exercise, patients with cirrhosis had a prolonged QT interval and an impaired relaxation between electrical and mechanical systole, termed electromechanical dyssynchrony.^104^^,^^105^ However, although primary liver disease has been associated with altered cardiac myofilament proteins, collagens, and left ventricular myocardial stiffness, the effects of CLD on heart function remain less clear and requires additional investigation.^106^

Bile acids represent a potentially important connection between liver pathology and cardiac disease. As mentioned previously, bile acids levels are altered in patients with HF, and they have been shown to have negative inotropic effects on cardiomyocytes. Cardiomyocytes isolated from dogs that have undergone BDL were less responsive when challenged with isoprenaline.^107^ Elevated bile acids have been postulated to induce cardiomyocyte dysfunction through their effects on membrane currents.^108^ In line with this notion, bile acids have been shown to reduce β-adrenergic receptor numbers and density and membrane fluidity.^109^ Bile acids can also alter signaling in the heart. In a recent study, RNA-seq of hearts exposed to elevated systemic bile acid concentrations revealed up-regulation of hypoxic, apoptotic, and proinflammatory pathways.^110^ Furthermore, bile acid accumulation in cardiomyocytes can lead to mitochondrial DNA release and myocardial inflammation.^111^ Together, these data indicate the cardiotoxic role of liver-derived bile acids. Defining the relationship between specific bile acids and their receptors in the pathogenesis signaling in CLD.

#### Vasoplegia

Patients with advanced liver fibrosis or cirrhosis have an increased risk of perioperative mortality after both general and open-heart surgery.^112^^,^^113^ In patients with end-stage HF undergoing heart transplantation, the MELD-XI score, is an independent predictor of post-transplant mortality.^114^ Similarly, in HF patients undergoing LVAD implantation, those with higher MELD-XI scores had worse survival and increased postoperative bleeding and vasoplegia.^115^, ^116^, ^117^ The risk of vasoplegia may be explained by altered clearance of vasodilators and/or increased release of inflammatory cytokines caused by liver dysfunction. Biochemical studies indicate that liver impairment often does not normalize until at least 6 to 12 months after heart transplantation.^16^

#### Increased infection risk

Patients with HF are predisposed to infection-related hospitalizations and short-term mortality when compared with patients without HF.^118^, ^119^, ^120^ Notably, patients with chronic HF have elevated levels of proinflammatory cytokines, including TNFα, IL-1, and IL-6.^121^, ^122^, ^123^ However, the etiology of this systemic immune activation remains unclear. One proposed mechanism is that intestinal congestion permits bacterial translocation from the gut to the portal circulation. In support of this, patients with chronic HF have increased bowel wall thickness, intestinal permeability, and mucosal bacterial density when compared with healthy control subjects.^124^ Additionally, liver fibrosis impacts endothelial and KC function which impair bacterial clearance. In line with these concepts, patients with HF have increased serum levels of LPS.^125^, ^126^, ^127^ The chronic proinflammatory state coupled with the loss of the intestinal and hepatic barrier function likely contributes to the enhanced susceptibility to systemic infections observed in patients with RHF.

## Future and Translational Directions For CLD Research

### Models and approaches to make new discoveries

**Figure 2:**
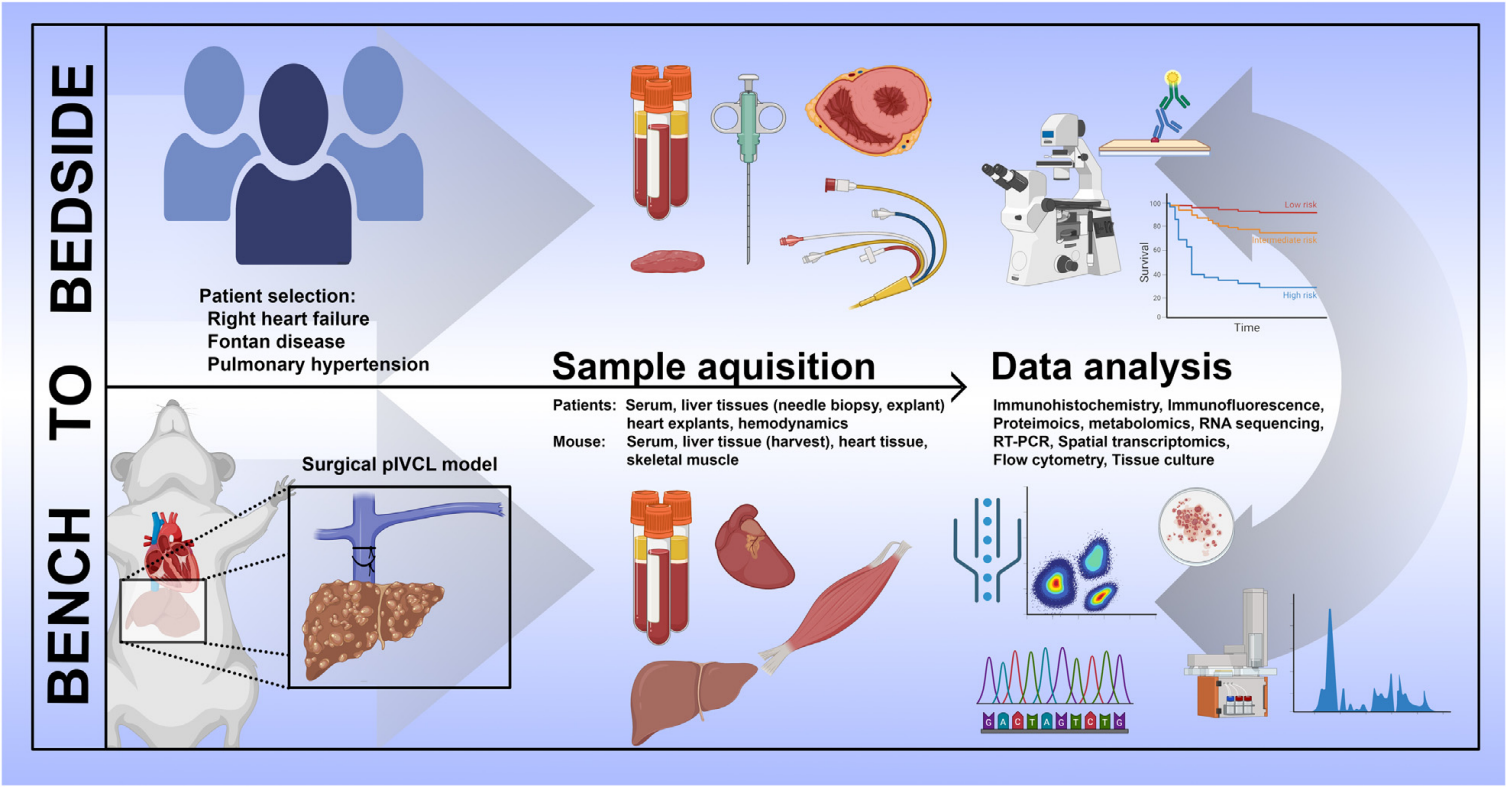
Translational Approaches to Define the Mechanisms and Consequences of Cardiogenic Liver Disease RT-PCR = reverse transcriptase polymerase chain reaction.

Compared with primary liver disease, studying CLD in patients can be challenging because of challenges with tissue availability and lack of disease awareness. To date, most studies in this area have relied on liver samples obtained from patients with end-stage HF undergoing evaluation for heart transplantation or LVAD surgery. Most of the samples are procured via a transjugular needle biopsy, limiting the amount of tissue that is available for downstream analysis. Although similar barriers exist for tissue analysis in patients with FALD, they are more likely to undergo combined organ transplant, which makes provides additional liver tissue. The use of newer techniques such as spatial transcriptomics, single nuclear RNA-seq, and codex multiplex imaging will continue to facilitate the cellular and molecular analysis using small amounts of tissue (Figure 2).

One important limitation of patient samples is that mechanistic studies are challenging to conduct. Therefore, developing preclinical models that recapitulate features of CLD in humans will be necessary to advance the field. Our group and others have employed a surgical model in which a rodent’s IVC is partially ligated, which mimics the hepatic outflow obstruction seen in RHF. The histologic, cellular, and molecular features of this model recapitulate many aspects of human CLD.^7^ The advantage of this model is that it allows investigators to study the effect of venous congestion on liver pathology, independent of cardiac dysfunction. However, because cardiac-derived factors may also contribute to CLD, combining pIVCL with HF models and/or utilizing pulmonary artery banding to produce RHF may also be useful. Using larger animal models of FALD and CLD is also an area of interest for translational studies.

### Translation of findings to the clinical sphere

#### Biomarkers

Traditional biomarkers of CLD commonly include elevated biochemical markers indicative of hepatic congestion and cholestasis. Modest elevations of AST, ALT, and total bilirubin have been associated with poorer survival in patients with HF and FALD.^128^^,^^129^ To identify other factors that may predict CLD, we utilized multiplex protein assays and mass spectrometry to identify elevated serum concentrations of sCD163, CXCL12, and bile acids as markers of liver pathology in patients with end-stage HF. Moreover, these biomarkers also predicted worse patient survival. Proteomic analysis of serum from patients with FALD also recently demonstrated that soluble CD44 concentrations in serum and liver correlated with liver fibrosis severity. CD44 expression was also found in the liver following pIVCL, and blockade of CD44 was found to attenuate liver fibrosis.^130^ These studies highlight the ability of circulating molecules to help identify potential mechanisms that contribute to RHF-associated CLD or vice versa.

#### Molecular tissue analysis

To use of transcriptional, translational, and epigenetic profiles in CLD has the potential to identify molecular pathways that contribute to CLD pathogenesis. Transcriptomic and single nuclear sequencing have recently employed to analyze liver samples from FALD as discussed in the previous text.^29^^,^^60^ However, because of the complex zonation of the liver, the use of spatial transcriptomic and proteomic approaches will also provide complementary information. Metabolomic analyses have also been pursued and are likely to provide additional clues about the mechanisms and consequences of CLD (Figure 2).

To date most investigation of CLD has focused on how HF impacts liver remodeling. While this remains important, additional studies are needed to assess the impact of CLD on other tissues. The pIVCL model is well situated to unravel the effects of isolated congestive liver disease on other tissues including the heart, skeletal muscle, and adipose. In addition, combining this model with other forms of HF would allow for the investigation of how liver disease impacts HF progression.

#### Modification of key pathways in CLD

Historically the only treatment for CLD is to treat the underlying cardiac disease. Whether the liver can be targeted to impact morbidity and mortality in patients with congestive liver pathology remains an open question. When considering therapeutic strategies, both liver dysfunction/fibrosis and the systemic effects of liver derangements are relevant to outcomes. For example, targeting liver fibrosis with antifibrotic agents or HSC modulators might improve liver function and/or restore hepatic clearance of toxins. Ultimately, more research is needed to identify the cellular and molecular effectors that are the primary drivers of CLD. To this end, our group recently found that LCN2, a lipocalin that has inflammatory and fibrotic properties, was highly expressed in macrophage aggregates and HPCs localized to regions of HSC activation.^7^ Whether LCN2 will serve as a useful biomarker or disease target remains to be tested. Macrophage modulation may also be a useful approach to improve liver function in RHF. As discussed earlier, KCs and MdMs change in number and morphology in the congested liver, particularly in regions of the liver with pathologic remodeling. Whether immunomodulation of macrophage function can be employed to influence the progression of CLD will require a deeper understanding of macrophage activation states in the congested liver. The liver also serves as a central hub of systemic metabolism. Therefore, further investigation of the metabolic implications of CLD will be required. The liver controls the systemic levels of several metabolites and nutrients including glucose, ketone bodies, fatty acids, lipoproteins, and bile acids, all of which can influence the function of tissues around the body. Whether hepatic metabolites or metabolic regulation can be leveraged therapeutically remains to be determined.

### Interplay between CLD and other liver pathologies

Patients with other primarily liver diseases, including MASLD and alcoholic liver disease, are enriched in the HF population. Thus, the interplay between metabolic, alcoholic, and congestive stress may produce liver pathology with unique features. In the case of MASLD, it is known that these patients have an increased incidence of coronary artery disease, HF, and cardiac arrhythmias despite adjusting for common cardiometabolic risk factors (see the other compendium in this issue for more discussion). Furthermore, the risk of HF directly correlates with the degree of liver disease and fibrosis.^131^, ^132^, ^133^, ^134^, ^135^, ^136^ The implications of concomitant CLD and MASLD are not known but are clinically relevant, especially given the increasing burden of obesity and MASLD. While alcohol abstinence is recommended for those with HF, excessive alcohol use is also common in this patient population. Therefore, dissecting the interplay between alcohol and CLD is another unmet need in this field.

## Conclusions

CLD is a complex and heterogenous disease that has been observed in patients with RHF for over a century. More recently, human transcriptomics and surgical rodent models of hepatic congestion have revealed new insights into disease pathogenesis. Based on current data, hepatic congestion appears to be both necessary and sufficient to cause pathologic liver remodeling. Although there have been significant advances in our understanding of CLD over the past couple of years, further work is needed to delineate the mechanisms of liver pathology and the systemic effects of CLD. Ultimately, uncovering molecular pathways of crosstalk between heart and liver will aid in the discovery of novel therapeutic targets for patients with CLD (Central Illustration).

**Central Illustration:**
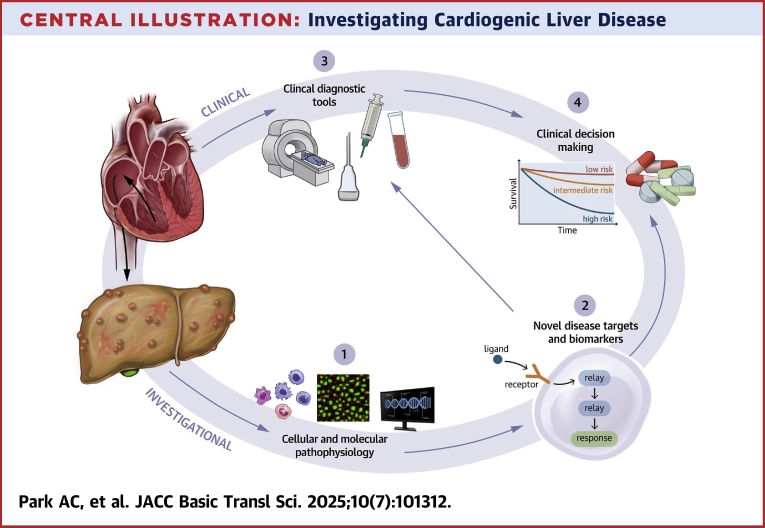
Investigating Cardiogenic Liver Disease (1) Characterization of cellular and molecular pathology: tissue histology and immunofluorescence, flow cytometry, RNA sequencing, proteomics, tissue cultures. (2) Data analysis and discovery of novel disease targets and biomarkers. (3) Collection of clinical data, imaging, and tissue samples. (4) Validation of novel biomarkers and disease pathways, clinical trials, and implementation of disease modifying drugs.

## Funding Support and Author Disclosures

The authors have reported that they have no relationships relevant to the contents of this paper to disclose.
